# Salvinorin A decreases mortality and improves neurological outcome in a neonatal mouse hypoxia model

**Published:** 2014-10-08

**Authors:** Chunhua Chen, Xu Cui, Felipe Matsunaga, Jingyuan Ma, Nan Ma, Ted Abel, Renyu Liu

**Affiliations:** 1 Department of Anesthesiology and Critical Care, Perelman School of Medicine, University of Pennsylvania, Philadelphia, PA 19104; 2 Department of Anesthesiology, Beijing Tongren Hospital, Capital Medical University, Beijing, China 100730; 3 Departent of Biology, University of Pennsylvania, Philadelphia, PA 19104

Neonatal hypoxic insults induce a variety of problems that include significant neonatal morbidity and long-term neurological deficits, such as motor deficits and learning disorders. Our previous study demonstrated that salvinorin A (SA), a non-nitrogenous selective kappa opioid agonist, protects the brain following ischemic/reperfusion injury in piglets. Here, we studied the neurological outcome after hypoxic insult in the presence and absence of SA pretreatment in neonatal mice. Eighty-three C57BL/6J mouse pups (postnatal day 1) were randomly divided into the following 3 groups: a control group (G1, n=11) received i.p. injection of DMSO (17%, same as in other groups); a hypoxia group (G2, n=46) received DMSO immediately before hypoxic insult; and a SA group (G3, n=26) received i.p. injection of 0.5 mg/kg SA in DMSO immediately before hypoxic insult. The hypoxic insult was induced by placing the pups in a sealed chamber with a mixture gas with 8% oxygen and 92% nitrogen for 120 minutes. Mortality rate in each group was determined. From postnatal day 2 to postnatal day 21, the pups were weighed and developmental motor behaviors (crawling, walking, running, head point and sniffing, sitting, rearing and eye opening) were observed for 3 minutes every day. Placing reflex, cliff aversion, negative geotaxis test, righting response and forelimb grasping tests were also performed. The first appearance of the neurodevelopmental milestone that continued for two consecutive days was determined. The long-term neurobehavioral effects of SA were observed at the age of 10–11 weeks by utilizing zero maze, Barnes maze and fear conditioning testing. Data were presented as % or mean ± SEM. SA significantly decreased the mortality from 70% to 38.5% in hypoxia group (*p*=0.0135). Meanwhile, body weight on postnatal day 2 and day 3 elevated in SA treated group (*p*=0.0318 and *p*=0.0221, respectively) compared to that in the hypoxia group. Furthermore, the SA group presented better performance of emergency of rearing at 18.7 ± 0.4 days compared to 19.9 ± 0.3 days in the hypoxia group (*p*=0.0252). Meanwhile, SA rescued hypoxia-induced delays in forelimb grasping, cliff aversion, righting response, eye opening and rearing activity. No statistically significant differences for the neurological outcomes tested in this study beyond 21 days were observed among groups. The reduction in mortality and improvement of the short-term neurological outcome by SA in a neonatal mice hypoxia model suggested its potential use as a medication to reduce hypoxia-induced neurological injury in the perinatal period.

## INTRODUCTION

Neonatal hypoxic-ischemic (HI) injury can induce high mortality and lifelong catastrophic neurologic and neuro-developmental deficits that include epilepsy, learning disabilities, and behavioral disorders (1). Unfortunately, no effective drug is available for managing HI-related neurological deficits. Therapeutic hypothermia along with supportive treatment is considered to be the only effective approach (2). However, well-controlled hypothermia can only be applied to highly selected patient populations in well-established facilities and it can only produce an outcome improvement of only about 30% in asphyxiated infants (3). It requires extensive training and can be dangerous (4). Thus, there is a significant medical need to develop a novel medication and/or easily manageable therapeutic strategy to reduce neuronal injury from cerebral HI in the perinatal period.

Salvinorin A (SA) is a highly selective and potent kappa opioid receptor (KOR) agonist extracted from Salvia divinorum, a plant that has been consumed by humans for several centuries (5). Using a piglet model, we demonstrated that SA administration before or after brain HI protects the brain from HI and can dilate cerebral vessels significantly, which was an important feature needed during brain hypoxia (6–8), indicating that SA could be a potential medication for brain protection for neonates. Unlike other opioid KOR agonists, SA does not produce frank dysphasia (9) and its intrinsic characteristics make it an alternative therapeutic candidate for various neurological conditions (10–12). These characteristics include: rapid onset, easy passage to the central nervous system, anti-nociceptive and sedative effects, no negative pathological changes in organs following prolonged or high dose exposure, and no respiratory depressive effect (10,12,13). Although the compound has been reported to have hallucinogenic activity, such effects are short-lived and do not induce significant blood pressure and heart rate changes or long term cognition impairment (14). In a recent human study, no persisting adverse effects related to SA were observed (15). Data from these clinical trials indicated that SA could potentially be used in humans safely for therapeutic purposes.

To demonstrate that SA could be potentially used in human subjects, similar effects have to be observed in a second species in addition to the findings in piglet. In the present study, we hypothesized that SA administration could improve outcomes in a neonatal mouse hypoxia model. The mouse model was chosen due to the availability of well-established neurobehavioral testing strategies and the capability to investigate both short-term and long-term neurological outcomes.

## MATERIALS AND PROCEDURES

Salvinorin A (purity ≥98%) was obtained from Apple Pharms (Asheville NC, USA). All other chemicals (reagent grade) were obtained from Sigma-Aldrich (St. Louis, MO, USA).

### Experimental Protocol

The protocol was approved by the Institutional Animal Care and Use Committee (IACUC) of the University of Pennsylvania and carried out in accordance with National Institutes of Health guidelines for the use of animals. C57BL/6J mice were purchased from The Jackson Laboratory (Bar Harbor, ME) and inbreed pups were used for subsequent experiments. Pups were housed under a 12-hour light/dark cycle after birth with free access to food and water throughout the study. Pups from different litters were divided into 3 groups randomly. Pups in the control group (n=11) received i.p. injection of 17% dimethyl sulfoxide (DMSO) without hypoxic insult; the hypoxia group (n=46) received i.p. injection of 17% DMSO before hypoxic insult; SA group (n=26) received i.p. injection of 0.5mg/kg SA in 17% DMSO before hypoxic insult. Pups of low weight (<1.2g) were excluded from the study. All the neurological tests were performed in a blinded manner.

### Hypoxic Insult

The hypoxic insult was induced on postnatal day 1. After i.p. injections, pups were put into a glass chamber in a water bath where the temperature was maintained at 37°C. The chamber was tightly closed and filled with 8% oxygen with balanced nitrogen. Following 120 minutes of hypoxic gas exposure, the chamber was opened and the pups were exposed to the air. Chest compressions and limb stretches were performed for up to 20 minutes to regain spontaneous breathing. The pups that successfully regained spontaneous breathing were then returned to their mothers after recovery for 30 minutes.

### Mortality Rate Determination

The neonate mouse will be considered as non-survival if no spontaneous breathing can be restored for 20 min resuscitation immediately after hypoxic insult. The mortality rate will be calculated as: survival rate = (number of mice in a group-number of non-survival)/number of mice in a group.

### Short-term developmental motor behavior observation

From postnatal day 2 to postnatal day 21, the pups were weighed and motor behavior observation was performed every day. Pups were observed individually in a plastic testing box. Each of the following behaviors was recorded for 3 minutes: crawling, walking, running, head point and sniffing, sitting, rearing and eye opening. After the observation, placing reflex, cliff aversion, negative geotaxis, righting response and forelimb grasping testing were performed. The first appearance of the behavior that continued for two consecutive days was recorded.

### Long-term neurobehavioral effects of SA

The open field test was performed to gauge locomotor activity on postnatal day 21 at which the mouse was considered juvenile. Mice were individually placed in a 41cm (L) × 41 cm (W) × 30cm (H) plastic box. The “central area” was defined as a 20.5cm × 20.5cm square in the center of the box. The rest of the area was defined as the “peripheral area” (see Figure 5A). The time each mouse spent on exploring the central and periphery parts and the number of rearing behaviors in each respective area were recorded for the first 5 minutes and 30 minutes (16).

At the age of 10–11 weeks, all mice were tested for several behavioral tasks in the order of: zero mazes, barnes maze and fear conditioning. These tasks were performed to examine the basal anxiety level, spontaneous locomotor activity, motor learning, spatial learning and associative memory of the mice. The mice were given 5 days for rest between each behavioral test.

1. *Zero Maze (anxiety-like behavior):* “Time in open” measures the anxiety level of mice due to their tendency to avoid open spaces. The anxiety was measured by recording the time spent in the open vs. enclosed space. Increased anxiety correlates to the decreased time in the open spaces. Open/Closed transitions measure the overall activity. Increased transitions equate to greater activity (17).
2. *Barnes Maze (spatial learning):* Barnes Maze measures the ability to learn with visual cues. “Time to Target” measures the time taken to find the target hole in an arena with 19 other holes and several visual cues. The Barnes Maze is easier to set-up and probably less stressful and a valid alternative to the Water Maze to study spatial memory (18). One of the advantages of the Barnes maze task was that it was not influenced by stress as much as other similar tasks and no strong aversive stimuli or deprivation were used.
3. *Fear Conditioning (memory):* Training trial freezing, short term contextual freezing, long term contextual freezing and cued trial freezing were recorded to examine and assess the memory deficits after hypoxia injury (19).

### Statistical analyses

Mortality of pups in different groups was compared using a 2×2 contingency table. P-value was calculated with the Fisher exact test. For motor behavior observation, the first days of appearance for each parameter were compared with one-way ANOVA followed by Turkey’s test. Statistical analyses were performed with Graph-Pad Prism (Version 5.0). Data were showed as mean ± SEM. A value of *p*<0.05 was considered to be statistically significant.

## RESULTS

### SA significantly decreased mortality

**Figure 1:**
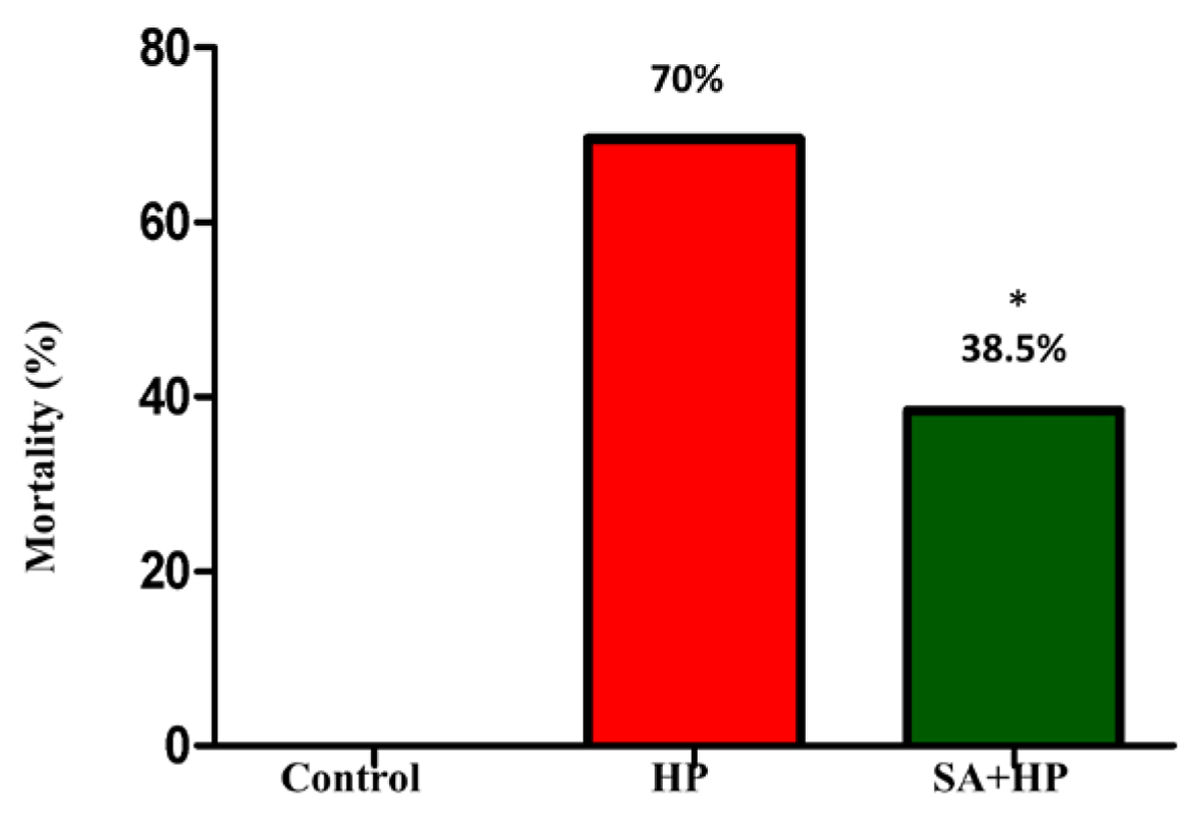
The mortality of different groups The mortality of HP and SA group was 70% and 38.5%, respectively. There was no death in the control group. The difference between SA and HP groups indicated that SA statistically decreased the mortality significantly (*p=0.014, SA+HP vs. HP. SA: Salvinorin A; HP: Hypoxia).

14 out of 46 pups in the hypoxia group and 16 out of 26 pups in the SA group survived after hypoxic injury. SA significantly decreased the mortality rate from 70% to 38.5% (*p*=0.014) (Figure 1).

### SA increased body weight on postnatal day 2 and day 3

**Figure 2:**
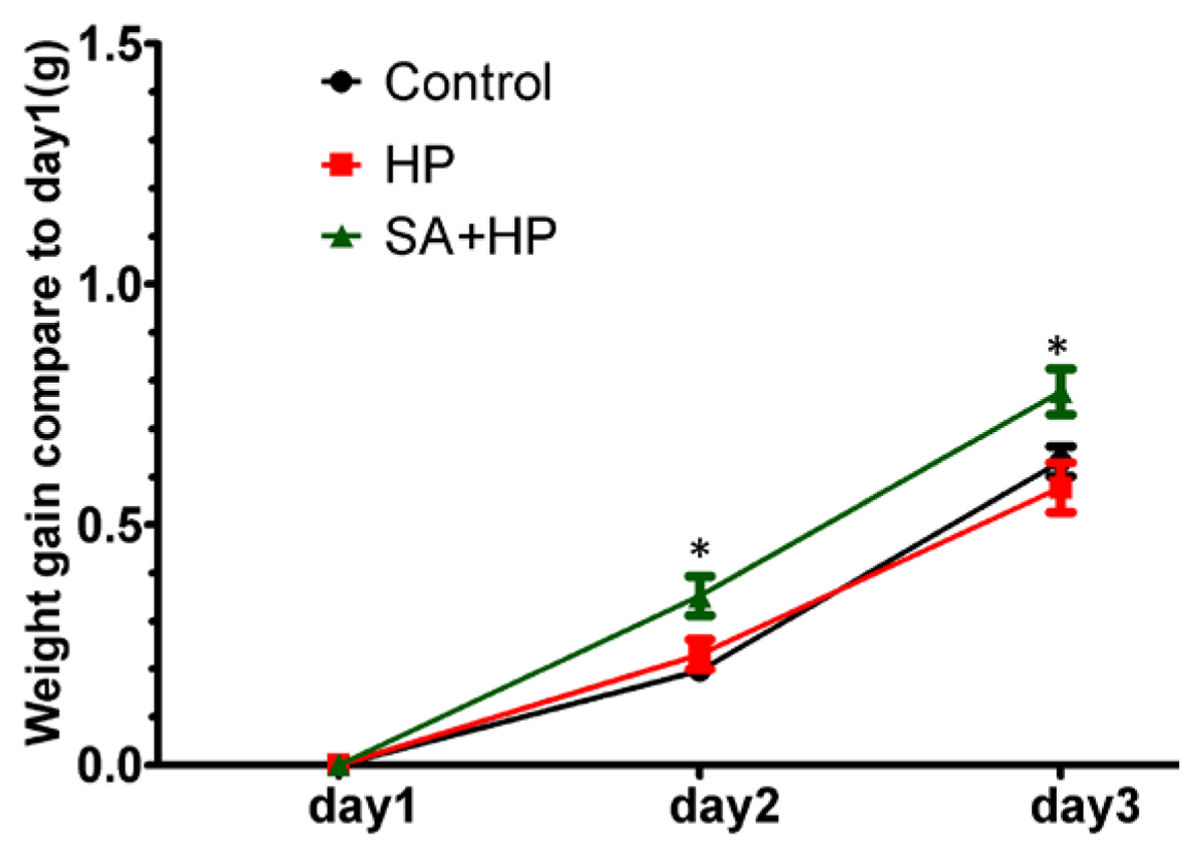
SA elevated the body weight gaining on postnatal day 2 and day 3 Body weight of pups in control, hypoxia, and SA treated groups on P1, P2, P3, P7, P14, and P21 were recorded in our study. SA treatment elevated the body weight gaining on postnatal day 2(P2) (p=0.0318) and postnatal day 3 (P3) (p=0.0221) compared to that in HP group. However, on P3, P7, P14 and P21, no significant difference was observed between different groups (data not shown) (*p<0.05 SA+HP vs. HP. P: postnatal; SA: Salvinorin A; HP: Hypoxia).

SA administration increased body weight compared with those in the hypoxia group on postnatal day 2 (P2) (*p*=0.0318) and day 3 (P3) (*p*=0.0221). However, no significant difference was observed among groups on P7, P14 and P21 (Figure 2).

### SA improved developmental outcomes

**Figure 3:**
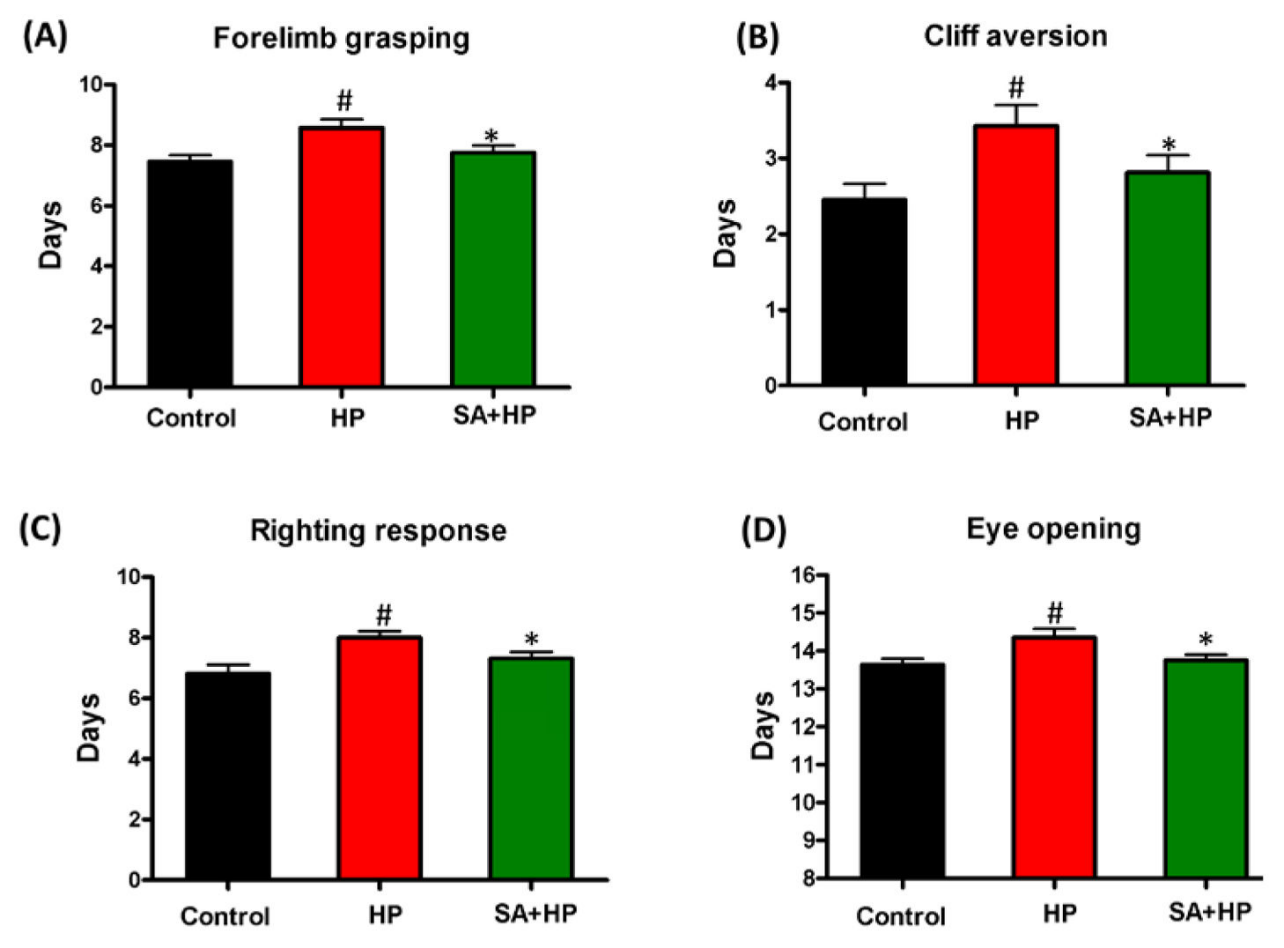
SA improved some of the developmental neurological outcomes Hypoxia induced significant delay in forelimb grasping (A), cliff aversion (B), righting response (C), and eye opening (D). SA rescued such hypoxia induced neurological outcomes significantly (# p<0.05 HP vs. Control; *p<0.05 SA+HP vs. HP. SA: Salvinorin A; HP: Hypoxia).

Pups in the hypoxia group showed delayed appearance of forelimb grasping (day 8.6 ± 1.0), cliff aversion (day 3.4 ± 1.0), righting response (day 8 ± 0.8) and eye opening (day 14.4 ± 0.8). SA administration prevented these delays (day 7.8 ± 0.9, 2.8 ± 0.9, 7.3 ± 0.9, and 13.8 ± 0.6 respectively) (Figure 3).

**Figure 4:**
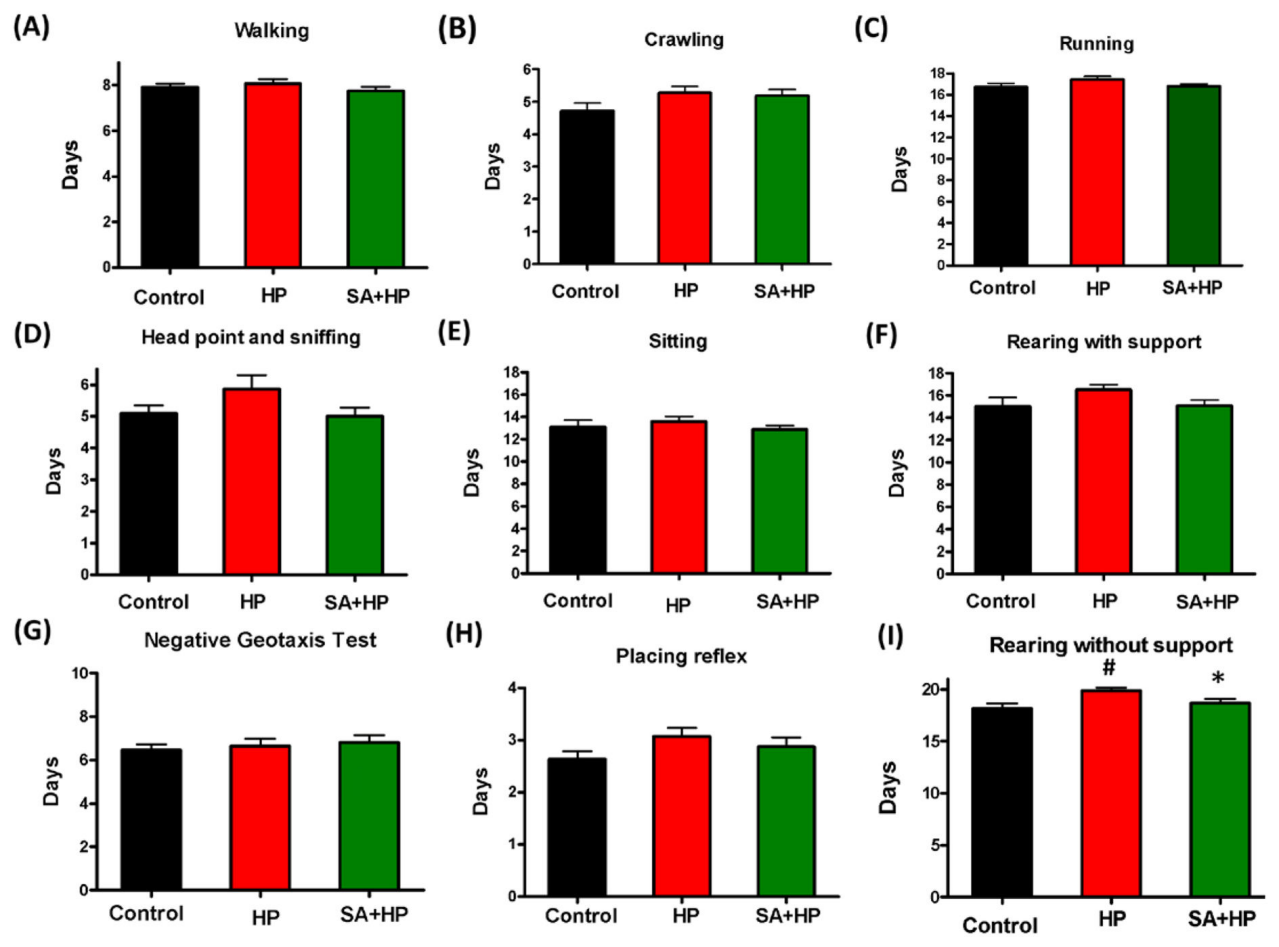
SA showed no statistical improvement on some of the development parameters There are no statistical significance on some of the development parameters such as walking (A), crawling (B), running (C), head point and sniffing (D), sitting (E), rearing with support (F), negative geotaxis test (G) and placing reflex (H), though hypoxic pups also showed delayed appearance compared to control and SA groups. Hypoxia pups showed delayed appearance of rearing without support at day 19.86±0.29 compared to day 18.18±0.4435 in control group. Hypoxic pups treated with SA presented better performance than hypoxic pups at day 18.69±0.3843(I), which is similar to the control group (# p<0.05 HP vs. Control; * p<0.05 SA+HP vs. HP. SA: Salvinorin A; HP: Hypoxia).

Hypoxic pups showed delayed appearance of rearing without support (Figure 4I) at day 19.9±0.3 compared to day 18.2 ± 0.4 in control group (*p*=0.0034). Hypoxic pups with SA administration presented significant improved performance than hypoxic pups at day 18.7±0.4 (*p*=0.0252), which is similar to the control group. However, there was no statistical significance with other developmental parameters such as crawling (Figure 4A), walking (Figure 4B), running (Figure 4C), head point and sniffing (Figure 4D), sitting (Figure 4E), rearing with support (Figure 4F) negative geotaxis test (Figure 4G) and placing reflex (Figure 4H), though hypoxic pups also showed delayed appearance compared to both the control and SA groups.

### SA did not change long-term neurobehavioral outcome

**Figure 5:**
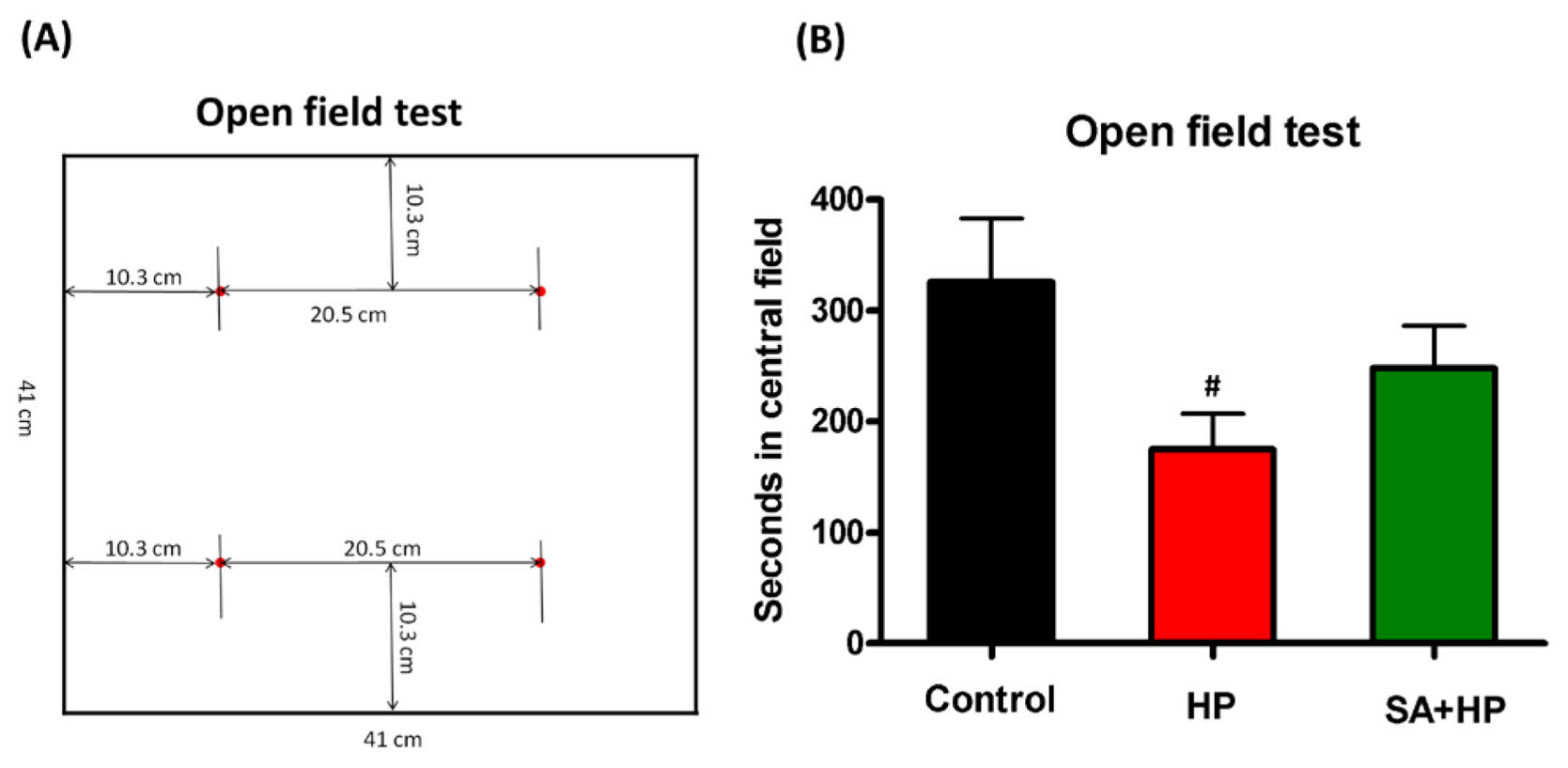
SA did not improve the outcome of open field test significantly (A) The left figure showed the sketch of the open field test. Mice were individually placed in a 41cm (L) × 41 cm (W) × 30cm (H) plastic box. “Central part of the area” was defined as a 20.5cm × 20.5cm square in the center of the box. The rest of the area was defined as “peripheral part of the area”. The time each mouse spent on exploring the central part and periphery part of the area and the number of rearing behavior were recorded respectively in the first 5 minutes and 30 minutes. (B) The seconds in the central field was recorded in open field test at P21 which showed that HP significantly decreased the seconds compared to the control and SA administration increased seconds in the central filed after hypoxia, but the difference was not statistically significant (# p<0.05 HP vs. Control. P: postnatal; SA: Salvinorin A; HP: Hypoxia).

**Figure 6:**
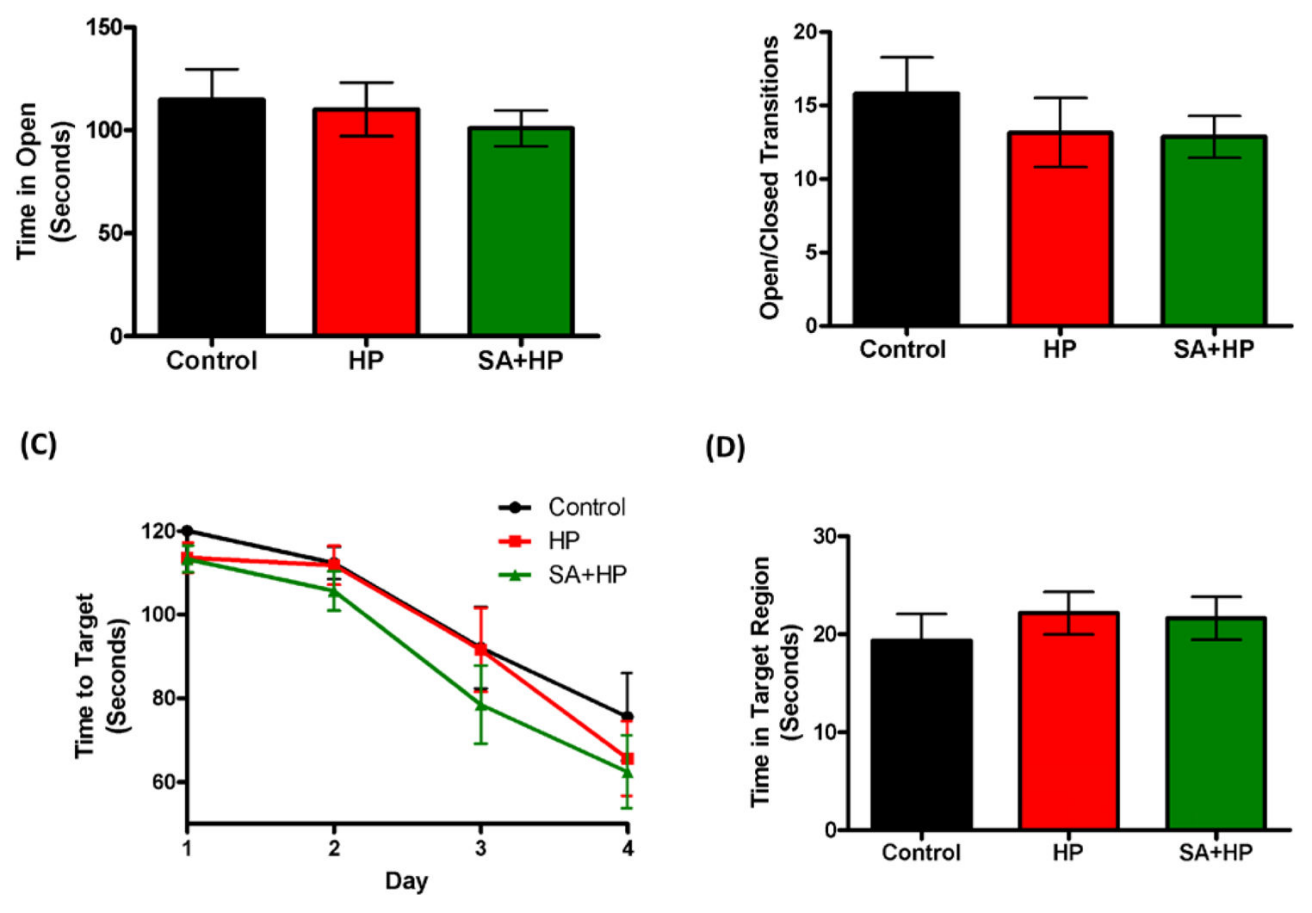
SA improved rearing activity at P21 significantly Rearing test at P21 in the first 5 min and 30 min was impaired by hypoxia and such impairment was not observed with SA administration which was similar to the control group, indicating that SA could improve some of the hypoxia induced long term neurological deficit (# p<0.05 HP vs. Control; * p<0.05 SA+HP vs. HP. P: postnatal; SA: Salvinorin A; HP: Hypoxia).

Although mice in SA group spent more time in the central area during open field testing at P21, this difference was not statistically significant (Figure 5). Meanwhile, the rearing test at P21 in the first 5 minutes and 30 minutes was impaired after hypoxia. This impairment was not observed with SA administration, similar to that in the control group, suggesting that SA could improve hypoxia induced the neurological outcomes (*p*=0.0167 and *p*=0.0203, respectively) (Figure 6).

**Figure 7:**
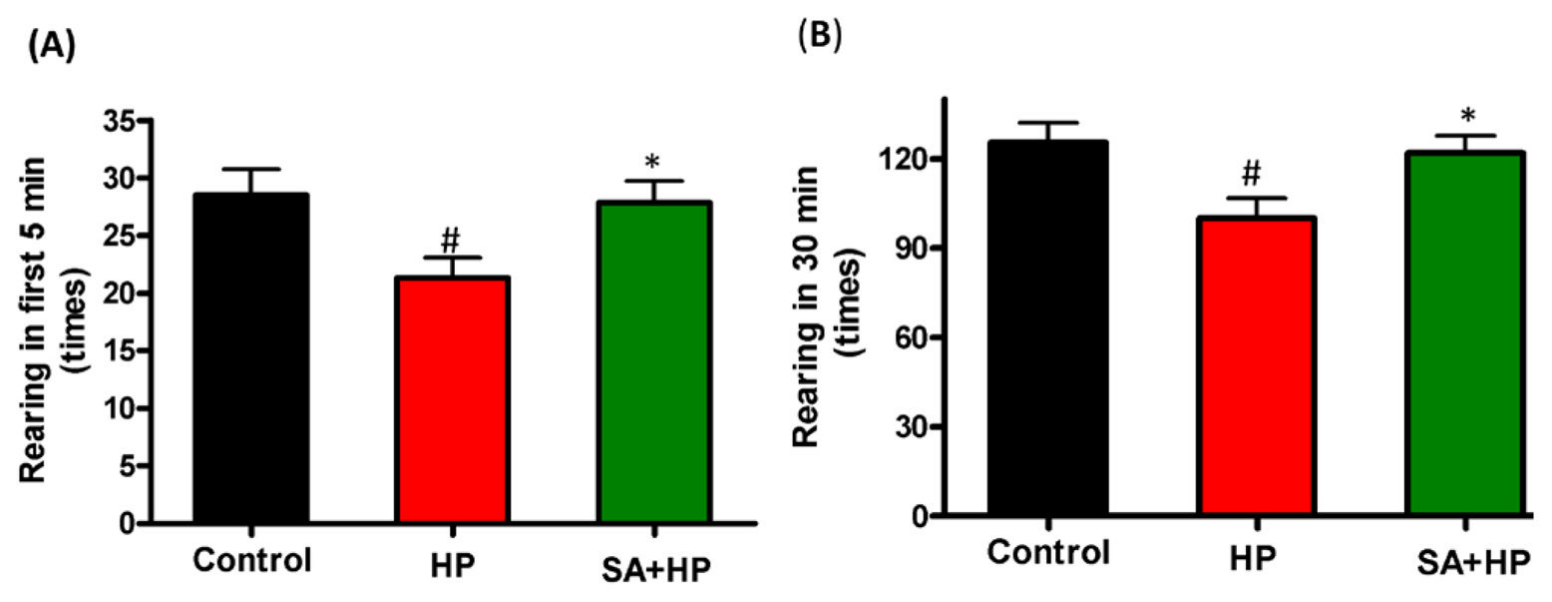
SA did not improve the long term anxiety level and spatial memory As for the elevated zero maze to detect the anxiety level and locomotor activity, there were no significant differences in the percentage of time spent in the open arm (A) and open/closed transitions (B) of the maze between SA administration and hypoxia alone groups. The Barnes maze task was used to assess spatial reference memory. In our study, there was no significant difference between the groups of the time to target (C) and time in target region (D). There was also no difference between the control and HP groups (SA: Salvinorin A; HP: Hypoxia).

The effects of SA on the anxiolytic properties after hypoxia were tested using an elevated zero maze. There were no significant differences in the percentage of time spent in the open arm and open/closed transitions of the maze between SA and hypoxia groups (Figure 7A, B).

**Figure 8:**
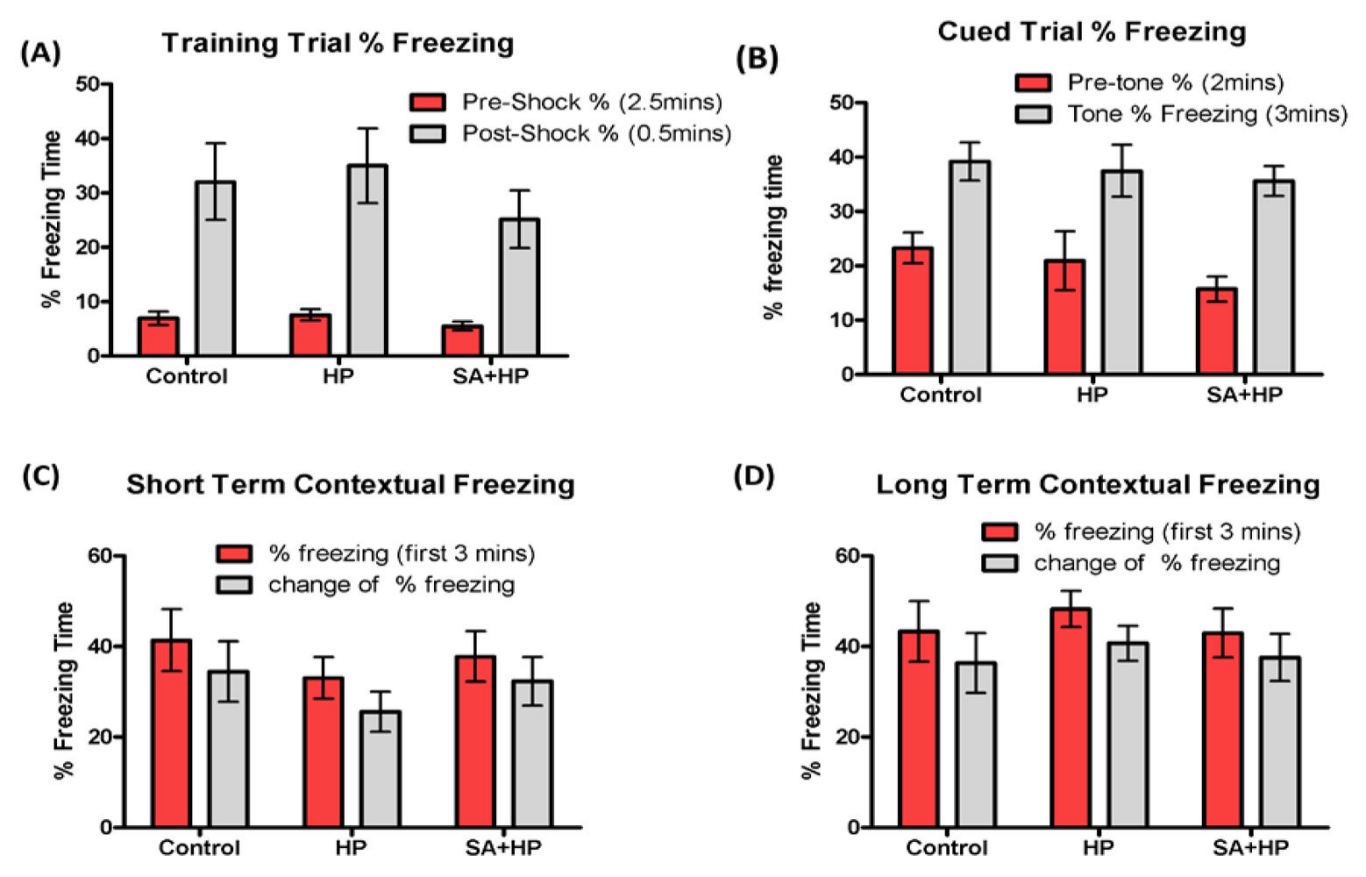
SA did not change the long term memory deficit All groups learned at similar rates and there was no significant difference in the long-term behavior of fear conditioning test. Control group showed no impairment compared to HP group in the training trial (A), cued trial (B), short term (C) and long term (D) contextual freezing behavior (p > 0.05). Pretreatment with SA did not change the fear memory compared with HP and Control mice (p > 0.05. SA: Salvinorin A; HP: Hypoxia).

The Barnes maze task was used to assess spatial memory deficits (20). However, there was no significant difference among groups (Figure 7C and D). Also, tests of fear conditioning showed that all groups learned at similar rates and no significant differences were observed among groups either (Figure 8, see above).

## DISCUSSION

In the current study, we found that SA administration before hypoxia reduces the mortality rate and improves several short term neurological development parameters within postnatal 21 days. However, no significant differences for neurological outcomes beyond 21 days were observed among groups. The findings in the present study were consistent with our previous study in piglets which revealed that SA could reduce HI induced injury (6–8).

### SA reduced mortality after hypoxia insult

The reduction of the mortality rate is one of the most striking findings in this study. While it could be attributed to the neurological protective effects of SA, it is possible that SA could offer protective effects in other vital organs like heart during hypoxic insults. It has been demonstrated that KOR agonist could protect heart from ischemia (21). Further well defined and targeted studies using SA as a potential therapeutics are needed for future studies. The mortality in the control group for this model was very high (70%), which is higher than that was reported using the modified Rice–Vannucci model (22,23). A possible explanation may be related to the use of the pups at postnatal day 1 in our study instead of postnatal day 10 which was used in the Rice-Vannucci model. However, the feasibility of using hypoxia instead of HI model on pups before postnatal day 10 was supported by previous studies (24,25). Due to the high mortality rate, we remained the design of the study relatively simple for neurological outcome observation only without dose-response studies and therapeutic time window investigation or mechanism studies.

### Short-term developmental outcome

Neurodevelopment was impaired by hypoxic insult as indicated by the deficits in neurological developmental behaviors and reflexes (righting, placing, negative geotaxis and cliff aversion). SA improved some of the developmental behaviors (forelimb grasping, eye opening and rearing without support) and some of the reflexes (righting and cliff aversion). Although some significant improvements were observed, SA did not improve all aspects of the developmental parameters that are related to various regions of the brain, which may be due to type II errors or different sensitivity to hypoxic injury in different brain regions.

### Long-term neurological outcome

To gauge brain function and the influences on emotional behaviors of SA in the long-term at 10–11 weeks (juvenile age), we chose zero mazes to measure the anxiety level, barnes maze to measure spatial and visual learning, and fear conditioning to measure associative memory. Interestingly, there was not any significant difference among groups. Most importantly, no difference of long-term neurological outcome was found between control and hypoxia group. This might be partially due to the fact that all the survived animals could be more resistant to hypoxic insult, thus there would be lack of sufficient injury to induce long term neurological outcome. For this reason, we did not exam histopathological outcome after the long term behavior outcome test. Although previous reports indicated that SA impaired long-term memory without affecting short-term memory (26), there wasn’t any memory or learning disability observed after SA administration in this study.

### Limitations

Firstly, both genders were used in this study. SA distribution and elimination differs among different genders and the kinetics are slower in female than in male monkeys (27), suggesting that gender specific studies should be planned in the future. Secondly, SA was administered before hypoxic insult which was less translational in most of the clinical scenarios except during difficult delivery which was highly predictable that perinatal hypoxia/ischemia is highly likely to occur. Thirdly, this is purely observational study and no mechanism is investigated.

### Future directions

The mechanism involved in the protective effects of SA on hypoxic neurons, astrocytes or other cell types still needs to be determined. While it is clear that SA activates KOR, several studies suggested that SA might induce brain metabolic changes in different brain regions through non-KOR dependent pathway (28,29). It is unclear whether such metabolic changes in different brain region could play important role in neurological protective effects.

Inhalation of SA is considered to be one of the efficient ways to obtain its psychoactive effects in humans and could induce psychoactive effects within seconds and last for minutes in a dose dependent manner (30). It would be important to find whether an ideal dose of SA could be identified that has the neurological protective effect with minimal or no psychoactive effects. Brain HI has very narrow therapeutic time window, thus, it is critical in the future studies to investigate the optimal time point of SA administration and define therapeutic time window for SA after HI insult. All these should be determined in a model with definitive long term neurological impairment in the absence of intervention instead of using the current model.

## CONCLUSION

In this study, the administration of SA is associated with improved neurological outcomes in several aspects and reduced mortality rate after hypoxic injury, suggesting that SA could be potentially served as an alternative treatment strategy for neonatal hypoxia.
